# Medical students working as health care assistants: a letter response in the COVID‐19 pandemic

**DOI:** 10.1111/tct.13219

**Published:** 2020-07-16

**Authors:** Henry Mitchell, Marco Coronelli, James Sanderson

**Affiliations:** ^1^ Department of Medicine and Dentistry Barts and The London School of Medicine and Dentistry London UK

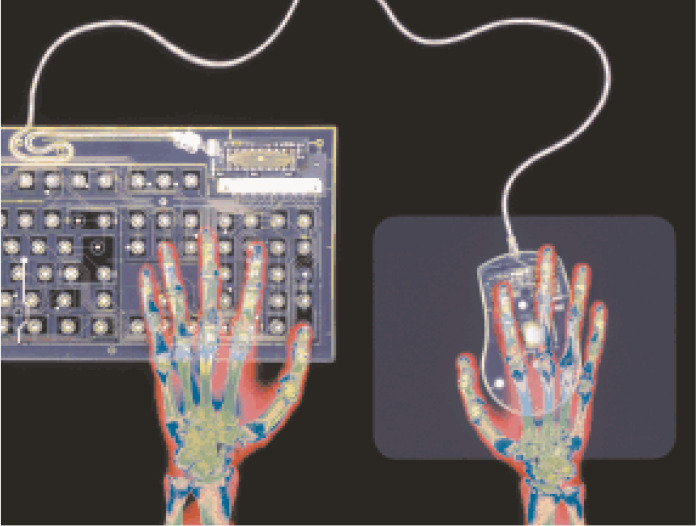

We read the study by Davison and Lindqvist regarding medical students working as health care assistants (HCAs) during the COVID‐19 pandemic with interest.^1^

As penultimate‐year students at Barts and The London School of Medicine and Dentistry, we had the opportunity to volunteer as HCAs when clinical placements were suspended. We undertook training that allowed us to directly support the multidisciplinary team (MDT) in its duties caring for ventilated patients on various wards. This differed from our previous experiences in a curriculum focused largely on shadowing clinicians.

As HCAs we performed basic nursing duties, such as toileting, mouth care and eye care. We assisted in manual handling, monitored patients and worked with therapists to rehabilitate patients’ swallowing, speech and mobility. In assisting with care, we have expanded our understanding of many different hospital roles, and of how the MDT works synergistically to accelerate patient recovery.^2^ Before this experience, we were not made aware in our curriculum of some of these interventions, as they do not constitute the traditional tasks of a doctor. Now, we have carried out these tasks and witnessed the difference that they can make to a patient's well‐being.

Working as HCAs has taught us caring skills that we can apply to learn more from our future clinical placements. The experience will influence our interactions with members of the MDT and help us to collaborate when managing patients as future clinicians. We therefore support the inclusion of a 3‐day HCA placement in medical training to enrich the shadowing experience.
